# Konsensusstatement der Migräne- und Kopfschmerzgesellschaften (DMKG, ÖKSG & SKG) zur Therapiedauer der medikamentösen Migräneprophylaxe

**DOI:** 10.1007/s00482-022-00671-9

**Published:** 2022-10-26

**Authors:** Gudrun Goßrau, Stefanie Förderreuther, Ruth Ruscheweyh, Victoria Ruschil, Till Sprenger, David Lewis, Katharina Kamm, Tobias Freilinger, Lars Neeb, Volker Malzacher, Uwe Meier, Klaus Gehring, Torsten Kraya, Thomas Dresler, Christoph J. Schankin, Andreas R. Gantenbein, Gregor Brössner, Karin Zebenholzer, Hans-Christoph Diener, Charly Gaul, Tim P. Jürgens

**Affiliations:** 1 Kopfschmerzambulanz, Universitätsschmerzcentrum, Medizinische Fakultät der TU Dresden, Universitätsklinikum Dresden, Fetscherstr. 74, 01307 Dresden, Deutschland; 2 Neurologische Klinik und Poliklinik, Ludwig-Maximilians-Universität München, München, Deutschland; 3 Deutsche Migräne- und Kopfschmerzgesellschaft, Frankfurt, Deutschland; 4 Klinik für Psychosomatik und Psychotherapie, Technische Universität München, München, Deutschland; 5 Abteilung Neurologie mit Schwerpunkt Epileptologie, Universitätsklinikum Tübingen, Tübingen, Deutschland; 6 Deutsche Klinik für Diagnostik, DKD Helios Klinik Wiesbaden, Wiesbaden, Deutschland; 7 LEWIS Neurologie, Stuttgart, Deutschland; 8 Klinik für Neurologie, Klinikum Passau, Passau, Deutschland; 9 Helios Global Health, Berlin, Deutschland; 10 Charité – Universitätsmedizin Berlin, corporate member of Freie Universität Berlin and Humboldt Universität zu Berlin, Klinik und Hochschulambulanz für Neurologie, Berlin, Deutschland; 11 Neurozentrum Reutlingen, Reutlingen, Deutschland; 12 Berufsverband Deutscher Neurologen, Wulffstr. 8, 12165 Berlin, Deutschland; 13 Berufsverband Deutscher Nervenärzte, Wulffstr. 8, 12165 Berlin, Deutschland; 14 Neurologische Klinik, Krankenhaus Sankt Georg Leipzig, Leipzig, Deutschland; 15 Neurologische Klinik, Universitätsklinikum Halle-Saale, Halle-Saale, Deutschland; 16 Klinik für Psychiatrie und Psychotherapie, Tübingen Zentrum für seelische Gesundheit, Universitätsklinikum Tübingen, Tübingen, Deutschland; 17 LEAD Graduiertenschule & Forschungsnetzwerk, Tübingen, Tübingen, Deutschland; 18 Neurologische Klinik, Inselspital, Universitätsspital Bern, Universität Bern, Bern, Schweiz; 19 Universitätsspital Bern, Universität Bern, Bern, Schweiz; 20 Neurologie & Schmerz, ZURZACH Care, Bad Zurzach, Schweiz; 21 Praxis Neurologie am Untertor, Bülach, Schweiz; 22 Universitätsklinik für Neurologie, Medizinische Universität Innsbruck, Innsbruck, Österreich; 23 Universitätsklinik für Neurologie, Medizinische Universität Wien, Wien, Österreich; 24 Institut für Medizinische Informatik, Biometrie und Epidemiologie (IMIBE), Medizinische Fakultät, Universität Duisburg-Essen, Essen, Deutschland; 25 Kopfschmerzzentrum Frankfurt, Frankfurt, Deutschland; 26 Kopfschmerzzentrum Nordost, Neurologische Klinik und Poliklinik, Universitätsklinik Rostock, Rostock, Deutschland; 27 Neurologische Klinik, KMG Krankenhaus Güstrow, Güstrow, Deutschland

**Keywords:** Migränetherapie, Migräne, Therapieempfehlung, Migräneerkrankung, Migränekopfschmerz, Migraine therapy, Migraine, Therapy guideline, Migraine disorder, Migraine headache

## Abstract

Migräne ist die häufigste neurologische Erkrankung und kann mit einem hohen Grad der Beeinträchtigung einhergehen. Neben nichtmedikamentösen Ansätzen zur Reduktion der Migränefrequenz stehen medikamentöse Migräneprophylaxen zur Verfügung. Zur Indikation und Anwendung liegen evidenzbasierte Leitlinien der Deutschen Migräne- und Kopfschmerzgesellschaft (DMKG) und Deutschen Gesellschaft für Neurologie (DGN), Österreichischen Kopfschmerzgesellschaft (ÖKSG) sowie Schweizer Kopfwehgesellschaft (SKG) vor. Für therapierelevante Fragen wie z. B. der notwendigen und sinnvollen Dauer einer medikamentösen Migräneprophylaxe können aus bisherigen Studiendaten keine Rückschlüsse gezogen werden. Ziel dieser Übersicht ist die Formulierung von Empfehlungen zur Therapiedauer bei wirksamer medikamentöser Migräneprophylaxe, die die aktuelle Datenlage und – bei fehlenden Daten – die Expertenmeinungen berücksichtigen.

**Zusatzmaterial online:**

Die Online-Version dieses Beitrags (10.1007/s00482-022-00671-9) enthält die AGREE Reporting Checkliste.

## Abstract (en)

Migraine is the most common neurological disorder and can be associated with a high degree of disability. In addition to non-pharmacological approaches to reduce migraine frequency, pharmacological migraine preventatives are available. Evidence-based guidelines from the German Migraine and Headache Society (DMKG), and German Society for Neurology (DGN), Austrian Headache Society (ÖKSG), and Swiss Headache Society (SKG) are available for indication and application. For therapy-relevant questions such as the duration of a pharmacological migraine prevention, no conclusions can be drawn from currently available study data. The aim of this review is to present a therapy consensus statement that integrates the current data situation and, where data are lacking, expert opinions. The resulting current recommendations on the duration of therapy for pharmacological migraine prophylaxis are shown here.

## Empfehlung

Die folgenden Empfehlungen für die klinische Praxis stellen eine Experteneinschätzung basierend auf der aktuellen Datenlage dar. Bei der Therapieplanung sind zusätzlich die Vorgaben der Fachinformation (also der Zulassungstext) und die jeweils aktuell gültigen nationalen Regelungen zur Erstattungsfähigkeit zu berücksichtigen.

Für jede medikamentöse Migräneprophylaxe muss im Verlauf die Indikation geprüft werden. Bislang wurde für die Dauer einer medikamentösen Migräneprophylaxe global eine Zeitspanne von 6–12 Monaten empfohlen. Ableitend aus der aktuellen Literatur wird die Individualisierung der Prophylaxedauer befürwortet. Dabei ist insbesondere für schwer betroffene Patienten (Patienten mit hoher migränebedingter Beeinträchtigung der Alltagsfunktion, chronischer Migräne (cM), nach oder bei Vorliegen von MOH (Medication Overuse Headache), psychischer Begleiterkrankung, chronischer Schmerzerkrankung) eine kontinuierliche Therapiedauer von 12 bis 24 Monaten zu ermöglichen. Die optimale Dauer einer Migräneprophylaxe ist nicht bekannt. Das gilt sowohl für spezifische als auch für unspezifische Substanzen der Prophylaxe. Über mögliche krankheitsmodifizierende Effekte von Migräneprophylaxen ist bisher nichts bekannt.

Grundsätzlich ist nach einem Absetzversuch erneut die Indikation zur medikamentösen Migräneprophylaxe zu prüfen, bevor die zuvor erfolgreiche pharmakologische Prophylaxe wieder aufgenommen wird.

Um eine maximale Wirksamkeit der Prophylaxe zu erreichen, sollte diese in eine optimierte Akuttherapie eingebettet sein und kombiniert mit nichtmedikamentösen Therapieoptionen verabreicht werden [1].

Wir empfehlen unter Voraussetzung einer ausreichenden Therapietreue des Patienten:

- für Patienten mit niedrigfrequenter episodischer Migräne (< 8 monatliche Migränetage; MMDs) zu Behandlungsbeginn, kürzerer Erkrankungsdauer und ohne komorbide Erkrankungen wie Depression, Angststörung oder chronische Schmerzerkrankung: 6 bis 12 Monate Therapiedauer bei klinisch signifikanter Reduktion der Migräne (d. h. ≥ 50 % Reduktion MMDs, ≥ 30 % Reduktion Migraine Disability Assessment Score (MIDAS), ≥ 5 Punkte Reduktion Headache Impact Test‑6 [HIT‑6]), bevor ein Absetzen der Migräneprophylaxe erfolgt
- für Patienten mit längerer Migräneanamnese, hochfrequenter episodischer (≥ 8 MMDs) oder chronischer Migräne zu Behandlungsbeginn und Begleiterkrankungen wie Depression, Angststörung oder chronische Schmerzerkrankung: mindestens 12 bis 24 Monate Therapiedauer bei klinisch signifikanter Reduktion der Migräne (d. h. ≥ 30 bis ≥ 50 % Reduktion MMDs, ≥ 30 % Reduktion MIDAS, ≥ 5 Punkte Reduktion HIT‑6), bevor ein Absetzen der Migräneprophylaxe erfolgt

## Hintergrund

Migräne ist eine häufige neurologische Erkrankung, die durch wiederkehrende, behindernde Kopfschmerzattacken, vegetative Begleitsymptome sowie eine mögliche Aura gekennzeichnet ist. Die Ätiologie ist genetisch multifaktoriell, wobei seltene monogene Varianten bekannt sind. Depression, Angststörungen, chronische Rückenschmerzen, Adipositas, und Schlafstörungen treten häufig komorbid auf. Die „Global Burden of Disease Study“ weist Migräne als zweithäufigste Ursache für ein Leben mit Behinderung aus, bei Frauen unter 50 Jahren steht Migräne dabei auf dem ersten Platz [2].

Der klinische Verlauf der Migräne variiert stark: von wenigen Migräneattacken im Leben bis zur chronischen Migräne, bei der Kopfschmerzen an 15 Tagen und mehr im Monat auftreten. Während der Großteil der Patienten eine niedrige Attackenfrequenz und niedrige Belastung zeigt, sind 10–20 % der Patienten mit hoher Attackenfrequenz für die hohe Beeinträchtigung und die Belastung des Gesundheitssystems verantwortlich [3]. Patienten mit cM zeigen demzufolge die umfangreichsten Einschränkungen der Alltagsfunktion, lange Zeiten der Arbeitsunfähigkeit und höhere gesundheitsbezogene Kosten [4].

Nach wie vor wird Migräne auch in Deutschland zu selten diagnostiziert und oft nicht adäquat behandelt [5].

In der Leitlinie zur Therapie der Migräne (DMKG und DGN, ÖKSG und SKG) werden neben den allgemein empfohlenen nichtmedikamentösen Ansätzen zur Reduktion der Migränefrequenz, wie z. B. Entspannungstechniken, Ausdauersport und Verhaltenstherapie, die Kriterien für den Beginn einer medikamentösen Migräneprophylaxe dargelegt (Tab. 1; [6]). Die Aktualisierung der Leitlinie wird im Herbst 2022 publiziert.

**Table 1:** 

Hoher Leidensdruck
Mindestens 4 Migränetage pro Monat oder lange oder schwer behandelbare Migräneattacken
Migräneattacken mit einschränkenden Aurasymptomen
Migräneattacken, die auf eine Akuttherapie nicht ansprechen
Risiko eines Medikamentenübergebrauchs

Dabei ist es Ziel der medikamentösen und nichtmedikamentösen Prophylaxe, die Frequenz, Intensität und Dauer von Migräneattacken zu reduzieren. Damit soll auch die Notwendigkeit der Einnahme von Akutmedikamenten zur Attackentherapie vermindert und der Entwicklung eines Kopfschmerzes bei Medikamentenübergebrauch (Medication Overuse Headache = MOH) vorgebeugt werden. Bei der episodischen Migräne (eM) wird eine Prophylaxe als wirksam beurteilt, wenn die Migränetage um mindestens 50 % reduziert werden und/oder die migränebedingte Alltagseinschränkung gemessen mit dem Headache Impact Test (HIT-6) um mindestens 5 Punkte oder dem Migraine Disability Assessment Score (MIDAS) um mindestens 30 % sinkt [7]. Nach Ergebnissen publizierter, randomisierter und kontrollierter Studien gilt bei der chronischen Migräne eine Reduktion um mindestens 50 % der Migränetage als Wirksamkeitsnachweis. In der Praxis kann jedoch auch eine Reduktion der Migränetage um mindestens 30 % als Therapieerfolg gelten. Insbesondere bei Patienten mit mehreren erfolglosen Therapieversuchen, Begleiterkrankungen wie Depression, Angststörungen oder chronischen Schmerzerkrankungen sowie MOH kann eine Reduktion der monatlichen Migränetage um mindestens 30 % einen entscheidenden Unterschied für die Alltagsfähigkeit und die migränebedingte Beeinträchtigung des Patienten bedeuten.

Die Wirkung der herkömmlichen, unspezifischen Substanzen zur Prophylaxe der eM, wie der Betablocker Metoprolol und Propranolol, des Kalziumantagonisten Flunarizin, der Antikonvulsiva Topiramat und Valproat sowie des Trizyklikums Amitriptylin sind durch randomisierte kontrollierte Studien (RCT) gut belegt. Bei cM mit oder ohne Übergebrauch von Schmerz- oder Migränemitteln sind Topiramat und OnabotulinumtoxinA gesichert wirksam [6].

Seit 2018 wurden durch die European Medicines Agency (EMA) in Europa vier neue, spezifische monoklonale Antikörper zur Prophylaxe der Migräne zugelassen (in der Reihenfolge der Zulassung: Erenumab, Galcanezumab, Fremanezumab und Eptinezumab). Sie richten sich gegen das Calcitonin Gene-related Peptide (CGRP) bzw. dessen Rezeptor (CGRP/(R)-AK). CGRP ist ein vasoaktives Neuropeptid, das in der Migräneattacke von afferenten trigeminalen Nervenfasern freigesetzt wird und pathophysiologisch als wichtigster Mediator einer Attacke gilt. Die migräneprophylaktische Wirkung der neuen spezifischen Substanzen ist ebenfalls durch zahlreiche RCTs sowohl für Patienten mit eM als auch mit cM belegt [8].

Während die migräneprophylaktische Wirkung einer Substanz in kontrollierten Studien gut belegt werden kann, ist es weit schwieriger, darüber zu entscheiden, wie lange eine wirksame Therapie durchgeführt werden soll. Schwere und der Verlauf einer Migräne können sich nicht nur von Patient zu Patient unterscheiden, sondern auch intraindividuell im Verlauf des Lebens erheblich variieren [9]. Es hat sich bewährt, im Verlauf einer Therapie die fortbestehende Indikation für eine wirksame Prophylaxe durch einen Absetzversuch oder die Reduktion der Dosis, oder die Verlängerung des Dosierungsintervalls zu überprüfen. Bei den bisher eingesetzten unspezifischen Migräneprophylaktika sind es jedoch vor allem unerwünschte Arzneimittelwirkungen oder eine unzureichende bzw. fehlende Wirkung, die dazu führen, dass die Therapie vorzeitig abgebrochen wird [10, 11].

Ziel dieser Übersicht ist es, die wissenschaftliche Evidenz für die angemessene Therapiedauer, den Zeitpunkt des Absetzens und den zu erwartenden Krankheitsverlauf nach dem Absetzen von medikamentösen Prophylaxen darzustellen. Dabei soll insbesondere beantwortet werden, ob eine wirkungsvolle Prophylaxe nach einem definierten Zeitfenster beendet werden sollte, welche zeitlichen Vorgaben dabei gelten sollen und wie hoch das Risiko für eine erneute Zunahme der Migränetage und der damit verbundenen Beeinträchtigung nach dem Absetzen einer Prophylaxe ist.

## Methode

Innerhalb der Expertenrunde, welche den Autoren des Konsensusstatements entspricht, wurden Fragen zur Therapiedauer bis zum sicheren Ansprechen auf die Migräneprophylaxe, zur Therapiedauer und zum Verlauf nach Therapieende von Migräneprophylaktika definiert.

Insbesondere für den Einsatz von CGRP/(R)-AKs haben diese Fragen aufgrund des migränespezifischen Wirkmechanismus und der bisher begrenzt verfügbaren Erfahrungen einen neuen Stellenwert (Tab. 2).

**Table 2:** 

Frage 1	Ist das Beenden/Absetzen einer medikamentösen Migräneprophylaxe sinnvoll?
Frage 2	Nach welcher Therapiedauer kann eine erfolgreiche Prophylaxe abgesetzt werden?
Frage 3	Welche Kriterien gelten für die Wiederaufnahme einer medikamentösen Prophylaxe?
Frage 4	Was muss bei einer langfristig eingesetzten medikamentösen Migräneprophylaxe beachtet werden?

Anhand dieser Fragen erfolgte bis April 2022 eine Literaturrecherche in Medline mit folgenden Begriffen: migraine, chronic migraine, discontinuation, treatment cessation, posttreatment period, amitriptyline, metoprolol, propranolol, topiramate, flunarizine, onabotulinumtoxinA, erenumab, galcanezumab und fremanezumab, eptinezumab. Diese Suche ergab 47.015 Ergebnisse. Die Mehrzahl der Publikationen wurde aufgrund fehlenden Bezugs zur Fragestellung exkludiert, 43 Publikationen wurden aufgenommen. Ebenso wurden weitere 11 relevante, den Experten bekannte Literaturstellen berücksichtigt, darunter 6 Therapieleitlinien, 2 Fachinformationen bzw. Dokumente des Bundesinstituts für Arzneimittel und Medizinprodukte (BfArM), 2 Reviews und eine klinische Studie. Anhand der Literatur und der klinischen Erfahrung der Experten wurden Vorschläge zum Vorgehen formuliert. Der Prozess erfolgte im formalen Konsensverfahren.

## Ergebnisse

### Daten zur Therapiedauer von Migräneprophylaxen und Verlauf nach Beenden der Medikation

Allgemein empfiehlt die deutsche Therapieleitlinie für Migräne eine Evaluation der Wirksamkeit ab ca. 2 Monaten nach Erreichen der tolerierten Höchstdosis für klassische unspezifische Migräneprophylaxen. Darüber hinaus wird empfohlen, eine erfolgreiche Migräneprophylaxe 6 bis 12 Monate nach Beginn auf ihre Notwendigkeit zu überprüfen [6].

#### Placebo

Eine Metaanalyse von Studien unspezifischer Migräneprophylaktika zeigte für Patienten, die Placebo erhielten, eine Reduktion der Kopfschmerzhäufigkeit nach 4 Behandlungswochen. Dieser Effekt hielt bis zur 12. Behandlungswoche an. In den Wochen 16, 20 und 24 stieg die Kopfschmerzfrequenz sukzessive wieder auf das Ausgangsniveau an [12].

#### Klassische unspezifische Prophylaxen

##### Betablocker: Metoprolol, Propranolol

RCTs zur Migräneprophylaxe untersuchten Metoprolol über einen Zeitraum von 4 bis 48 Wochen und Propranolol über 4 bis 64 Wochen [13]. Deutliche Effekte über einen Zeitraum ab 4 bis 8 (Metoprolol) bzw. 12 (Propranolol) Wochen gegenüber Placebo wurden gezeigt [13]. Da es keine Studien mit langfristiger Nachbeobachtung gibt, ist unklar, ob diese Effekte nach Absetzen der Betablocker stabil sind [14].

##### Topiramat

Das Konzept, die Notwendigkeit einer weiteren Behandlung 6–12 Monate nach Therapiebeginn zu überprüfen, beruht auf den Ergebnissen einer Studie mit Topiramat zur Migräneprophylaxe. Diese Studie untersuchte randomisiert und placebokontrolliert die Migränehäufigkeit nach dem Ende eines 6‑monatigen Behandlungszeitraums mit Topiramat [15]. Hier wurden Patienten mit Migräne nach einer 4‑ bis 8‑wöchigen Titrationsphase in einer 26-wöchigen offenen Phase mit Topiramat behandelt (50–200 mg/Tag) und darauffolgend zufällig einer 26-wöchigen Doppelblindphase zugewiesen, in der sie entweder die Topiramatdosis beibehielten oder zu Placebo wechselten. Zusammenfassend wurde nach dem Absetzen von Topiramat über alle Patienten hinweg ein anhaltender Nutzen festgestellt. Obwohl die Zahl der Migränetage unter Placebo zunahm, erreichte sie nach 26 Wochen nicht das Ausgangsniveau. Daraus schlussfolgerten die Autoren, dass Patienten mindestens 6 Monate lang behandelt werden sollten, wobei nach individueller Abwägung die Therapiedauer auf bis zu 12 Monaten verlängert werden kann.

##### Flunarizin

Studiendaten weisen darauf hin, dass die Wirkung von Flunarizin während eines viermonatigen Behandlungszeitraums zunahm. Die maximale Wirkung wurde nicht vor dem dritten Behandlungsmonat erreicht [16]. Dies könnte teilweise auf die Pharmakokinetik von Flunarizin zurückzuführen sein: Die Plasmakonzentration erreichte bei Gesunden mit täglicher Einnahme von Flunarizin erst 5–6 Wochen nach Beginn der Behandlung den Steady-State [17].

Für Flunarizin liegen heterogene Daten vor, die einerseits einen Langzeiteffekt von im Mittel 8 Monaten nach Therapieende auf die Migränefrequenz zeigen [18]. Andererseits konnte eine im Abschnitt „Valproat“ dargestellte Crossover-Studie mit Flunarizin keine Langzeitwirkung verifizieren [12, 16]. Weitere Studien belegen anhaltende Therapieeffekte 6 bzw. 7 Monate nach Therapieende bei der Mehrzahl der Patienten [19, 20]. Für Flunarizin ist laut Fachinformation eine Begrenzung der Therapiedauer geboten: „Lässt während der Behandlung der therapeutische Effekt nach, ist die Behandlung mit Flunarizin abzubrechen.“ „Selbst wenn die prophylaktische Weiterbehandlung erfolgreich war und gut vertragen wurde, sollte die Behandlung spätestens nach 6 Monaten beendet werden und nur bei Rückkehr der behandelten Symptome wieder eingesetzt werden.“ [21].

##### Valproat

Aufgrund der aktuellen Risikobewertung von Valproat durch das BfArM/das Bundesamt für Sicherheit im Gesundheitswesen (BASG) sollte es nicht mehr zur Migräneprophylaxe bei Frauen im empfängnisfähigen Alter eingesetzt werden [22]. Ältere Daten einer Crossover-Studie mit Valproat belegen jedoch nicht nur die Wirksamkeit in der Migräneprophylaxe, sondern weisen auch nach, dass die Zahl der Valproat-Responder in den letzten 4 Wochen eines 12-wöchigen aktiven Behandlungszeitraums auf 65 % (Responder im Gesamtzeitraum von 16 Wochen 50 %) im Vergleich zu 18 % bei Placebo anstieg [12, 23]. Sowohl in der Crossover-Studie mit Flunarizin [16] als auch in der Studie mit Valproat [23] kam es in der 4‑wöchigen Wash-out-Phase nach der Verumeinnahme zu einem Wiederanstieg der Migränetage bzw. -attacken, sodass hier kein über die Therapiedauer hinausgehender prophylaktischer Effekt gefunden wurde.

##### Amitriptylin

RCTs untersuchten Amitriptylin zur Migräneprophylaxe über einen Zeitraum von 4 bis 26 Wochen [12]. Die beste Wirkung wurde bei Amitriptylin in einer Studie erst nach 16 Wochen Therapie erreicht [24]. Daten zum Migräneverlauf nach Absetzen liegen nicht vor.

#### OnabotulinumtoxinA bei chronischer Migräne

Eine offene Untersuchung zum Therapieeffekt von OnabotulinumtoxinA zeigte, dass zwei Drittel der Patienten, die 3–6 Monate nach Beginn der Behandlung zu ≥ 50 % auf OnabotulinumtoxinA ansprachen, auch 3 Monate nach Absetzen des Medikaments weiterhin eine mindestens 50 %ige Reduktion der monatlichen Kopfschmerztage zeigten [25].

Eine durch die SARS-CoV2-Pandemie bedingte Injektionsverzögerung um im Mittel 52 Tage (SD ± 26) zeigte bei 80 retrospektiv untersuchten Patienten mit cM eine signifikante Zunahme der monatlichen Kopfschmerztage von 14,8 ± 7,7 auf 17,4 ± 8,8, sowie eine Zunahme der Analgetikaeinnahme und der Alltagseinschränkung (HIT-6-Fragebogen) [26]. Patienten mit > 30 % Zunahme der monatlichen Kopfschmerztage nach pandemiebedingtem Absetzen von OnabotulinumtoxinA wiesen eine längere Erkrankungsdauer, längeres Vorliegen eines MOH, häufigere Einnahme von Analgetika und eine höhere durchschnittliche Schmerzintensität auf [27, 28].

In einer offenen prospektiven Studie wurde bei OnabotulinumtoxinA-Respondern im zweiten Behandlungsjahr das Injektionsintervall auf vier Monate ausgedehnt [28]. 49/108 (45 %) der Patienten berichteten daraufhin eine Zunahme der Migränetage im Monat vier, woraufhin das Intervall wieder verkürzt wurde. Bei 10 (9,3 %) Patienten wurde die Therapie wegen mangelnden Ansprechens beendet, sechs Patienten hatten keine Attacken während der vier Monate und es wurde zugewartet; zwei Patienten erhielten ca. zweimal pro Jahr Injektionen, da es nach etwa sechs Monaten wieder zum Frequenzanstieg kam, und zwei Patienten hatten nicht mehr als zwei Attacken im Quartal über 18 Monate. Die übrigen 39 Patienten erhielten die Injektionen weiterhin alle vier Monate.

In einer weiteren Studie wurde bei Patienten mit cM und sehr gutem Ansprechen auf eine Therapie mit OnabotulinumtoxinA 6 Monate nach dem Absetzen bei 80 % keine klinische Verschlechterung bemerkt bzw. keine Notwendigkeit einer Wiederaufnahme der Prophylaxe gesehen. Patienten, die zur Baseline für mehr als 6 Monate an einem täglichen Kopfschmerz gelitten hatten, hatten nach einem Follow-up von 6 Monaten nach Absetzen ein höheres Rezidivrisiko als solche mit einem weniger als 6 Monate bestehenden täglichen Kopfschmerz. Auch Patienten mit einer höheren Anzahl an Behandlungszyklen bis zum positiven Ansprechen hatten ein höheres Rezidivrisiko als solche mit rascherem Ansprechen [29].

#### Spezifische Prophylaxemedikamente

##### CGRP/(R)-AKs: Erenumab, Galcanezumab, Fremanezumab Eptinezumab

Zusammenfassend zeigen die bisherigen Daten (10 Studien, 2171 Patienten), dass nach Absetzen einer erfolgreichen Migräneprophylaxe mit CGRP/(R)-AK bei der Mehrzahl der Patienten wieder eine schrittweise Zunahme der Migränefrequenz, der migränebedingten Alltagseinschränkungen und eine Zunahme des Bedarfs an Akutmedikation eintritt [30–39]. Die Studien variieren in der Methodik (retrospektiv versus prospektiv, kontrolliert versus „real-world“-Daten, Fallzahlen, Therapie- und Nachbeobachtungszeiträume, chronische versus episodische Migräne, mit versus ohne Medikamentenübergebrauch, untersuchte Wirkstoffe), was die Aussagekraft direkter Vergleiche einschränkt.

Die meisten Studien (7/10) untersuchten Effekte drei Monate nach Absetzen der CGRP/(R)-AK. Dabei zeigte sich in allen Studien im Mittel eine signifikante Zunahme der mittleren monatlichen MMDs im Vergleich zum Therapiezeitraum, nicht jedoch bei allen eine Zunahme der MMDs auf das Ausgangsniveau vor Therapiebeginn. So zeigte eine Studie mit Patienten mit cM und eM bereits 4 Wochen nach Absetzen der CGRP/(R)-AK-Therapie für 31 % der Patienten eine Zunahme der MMDs, welche zum erneuten Therapiebeginn führte [38]. Für Patienten mit cM, welche aufgrund der CGRP/(R)-AK-Therapie in eine prognostisch günstigere eM übergegangen waren, konnte gezeigt werden, dass fast 50 % im 3. Monat der Therapiepause einen Wiederanstieg auf mindestens 15 MMD berichteten. Ebenso ließ sich bei fast der Hälfte der Patienten, bei denen zu Beginn der Therapie ein Medikamentenübergebrauch bestand, im 3. Monat der Therapiepause ein erneuter Medikamentenübergebrauch feststellen [39]. In 3 der 10 Publikationen wurde berichtet, dass auch nach 3 Monaten Therapiepause die mittleren MMDs zwar signifikant zum letzten Therapiemonat anstiegen, jedoch weiterhin geringer waren als zur Baseline [30, 31, 40]. Hier sind Studiendaten zur gesundheitsbezogenen Lebensqualität während der Therapiepause mit CGRP/(R)-AK zu beachten, die bereits in Woche 8–16 nach Therapieende eine signifikante Zunahme der kopfschmerzbedingten Einschränkung der Alltagsaktivitäten und der Lebensqualität zeigen [37]. Ist eine Fortsetzung der Therapie mit CGRP/(R)-AK nötig, so belegen die dazu bislang publizierten Studien, dass die Mehrzahl der Patienten nach Wiederbeginn der CGRP/(R)-AK-Therapie genauso gut wie in der initialen Behandlung ansprechen [30, 32, 35].

Als Ursache für ungünstige Absetzeffekte müssen auch Nocebomechanismen diskutiert werden [41]. Dazu existieren bislang nur klinische Erfahrungen, jedoch keine wissenschaftlichen Daten. Im klinischen Alltag stellen Noceboeffekte eine relevante Einflussgröße dar, da Patienten einen Anstieg der Kopfschmerzfrequenz befürchten und erwarten.

In einigen Studien hielt der Therapieeffekt bei einem Teil der Patienten über das Ende der pharmakologischen Wirkung hinaus an. Dabei kann nicht entschieden werden, ob dies auf medikamentösen Langzeiteffekten oder anderen Faktoren wie natürlicher Fluktuationen im Verlauf der Migräneerkrankung oder der Wirkung begleitender Maßnahmen wie der nichtmedikamentösen Prophylaxe oder dem abnehmenden Einfluss psychischer Komorbiditäten beruht. Beispielsweise beschreiben Iannone et al. für 25 % der Patienten anhaltend reduzierte MMDs [30]. Ob hier ein krankheitsmodifizierender Effekt der CGRP/(R)-AK nach längerer Therapie vorliegt, ist Gegenstand der gegenwärtigen Forschung, bislang jedoch nicht belegt. Wertvoll sind Daten, die an Teilnehmern kontrollierter Studien erhoben wurden, da hier eine Abgrenzung zu Placeboeffekten möglich ist und ein größeres Patientenkollektiv untersucht wurde. Entsprechende Daten liegen aus der Evolve‑1 und -2-Studie mit Galcanezumab vor, in der Patienten bis zu 4 Monate nach Beenden der CGRP/(R)-AK-Therapie placebokontrolliert nachverfolgt wurden [34]. Interessanterweise unterscheidet sich die gesundheitsbezogene Lebensqualität und kopfschmerzbedingte Alltagseinschränkung in Monat 4 der Therapiepause nicht mehr zwischen der Placebogruppe, also der Gruppe, welche nie CGRP/(R)-AK erhielt, und der Therapiepausengruppe nach CGRP/(R)-AK-Therapie. In Letzterer kam es im Gruppenmittel zu einer Zunahme der MMDs, die jedoch nicht das Ausgangsniveau zur Baseline erreichte.

Als Prädiktoren für einen anhaltenden Effekt nach Therapieende konnten folgende Konstellationen identifiziert werden (3 Studien, 383 Patienten): guter Erfolg der Behandlung mit CGRP/(R)-AKs, niedrige MIDAS- und HIT-6-Scores (sowohl vor Beginn der Therapie als auch unter Therapie), niedrigere MMDs und weniger Schmerzmitteleinnahmetage vor Beginn der Therapie [30, 39] sowie (nur in einer Studie gezeigt) ein niedrigerer BMI und eine Migräne mit Aura [31]. Patienten mit vielen frustranen Therapieversuchen und hoher Anzahl von MMDs vor Beginn der Therapie hatten stattdessen ein höheres Risiko für einen frühen Rückfall (im 1. Monat nach Beendigung der Therapie) in eine chronische Migräne mit Medikamentenübergebrauch [31]. Bei der Betrachtung der Daten sollte berücksichtigt werden, dass in kontrollierte Studien Patienten aufgenommen wurden, die größtenteils weniger vorselektiert waren als gesetzlich versicherte Patienten, die nach den aktuellen Maßgaben zur Kostenerstattung in Deutschland erst spät Zugang zu den spezifischen Antikörpern erhalten. Grundsätzlich weisen jedoch auch die bislang vorliegenden „real-world“-Behandlungsdaten darauf hin, dass weniger schwer betroffene Patienten mit besserem Ansprechen auf die CGRP/(R)-AK-Therapie eine bessere Chance auf einen anhaltenden Effekt nach dem Absetzen haben.

## Weitere Aspekte der medikamentösen Prophylaxe bei Migräne

Bislang ist wissenschaftlich nicht geklärt, ob bestimmte Substanzen zur Migräneprophylaxe krankheitsmodifizierend wirken oder ausschließlich eine verlaufsmodulierende Wirkung haben.

Um die fortbestehende Indikation für eine Migräneprophylaxe und den Wirkeffekt einer Prophylaxe zu prüfen, ist das Absetzen der Medikation ein wichtiges Instrument. Als Anhaltspunkt wird in den entsprechenden Leitlinien empfohlen, die Behandlung 6–12 Monate nach Behandlungsbeginn auszusetzen [42]. In Ermangelung eindeutiger Erkenntnisse darüber, wie lange eine medikamentöse Migräneprophylaxe fortgesetzt werden sollte, muss die Entscheidung zum Zeitpunkt des Absetzversuchs individuell unter Einbeziehung des Patientenwunsches und der individuellen Lebensumstände des Patienten getroffen werden. Hier ist eine Abwägung der Verträglichkeit und Wirkung gegen die individuelle Beeinträchtigung durch die Migräne selbst, relevante Komorbiditäten einschließlich eines vorbestehenden Medikamentenübergebrauchs, das Ansprechen auf vorherige, auch nichtmedikamentöse Prophylaxen, den bisherigen Krankheitsverlauf auch in Hinsicht auf den Chronifizierungsgrad sowie die aktuelle Krankheitsaktivität erforderlich.

Das Absetzen kann abrupt oder ausschleichend (Betablocker, Topiramat) erfolgen. Die langsame Dosisreduktion ermöglicht die Bewertung, welche Dosierung zur Reduktion der Migränefrequenz ausreicht, auch wenn systematisch erhobene Erkenntnisse zu anderen Therapieintervallen als in den Zulassungsstudien nicht vorliegen.

### Absetzen der medikamentösen Migräneprophylaxe – wie gehen Patienten damit um?

Zahlreiche Studien zeigen, dass die Therapietreue bei der Einnahme unspezifischer Substanzen zur Migräneprophylaxe schlecht ist [10]. Grund sind vor allem unerwünschte Wirkungen der Therapie.

Bei guter Verträglichkeit der unspezifischen Substanzen oder wenn weitere gewünschte Wirkeffekte genutzt werden sollen, wie z. B. Schlafregulation und Stimmungsstabilisierung bei Depression unter Behandlung mit Amitriptylin oder Blutdrucksenkung unter Behandlung mit Betarezeptorenblockern, werden Patienten durchaus auch länger als 1 Jahr behandelt [43].

In Deutschland übernehmen die gesetzlichen Krankenkassen derzeit (Stand Juli 2022) die Kosten für eine Behandlung mit CGRP/(R)-AK für Patienten mit Migräne ab 4 MMDs nur, wenn alle unspezifischen Substanzklassen keine Wirkung gezeigt haben, nicht vertragen wurden oder kontraindiziert waren. In Österreich sind CGRP/(R)-AK ab 4 MMD zugelassen, mindestens 3 Versuche mit medikamentösen Prophylaxen müssen erfolglos verlaufen sein. Für die Schweiz gilt, dass 2 von 4 zugelassenen Substanzen eingesetzt worden sein müssen und dass die Behandlung ab 8 MMDs möglich ist. Die Vorbehandlung mit OnabotulinumtoxinA entfällt in der Schweiz, da dort keine Zulassung besteht. Diese Erstattungsrichtlinien haben zu einer erheblichen Selektion von besonders schwer betroffenen Patienten geführt. So ist nachvollziehbar, dass sie eine geplante Therapiepause der CGRP/(R)-AK als potenziell bedrohlich erleben. Es werden Ängste und Sorgen vor einer erneuten Zunahme der Migränehäufigkeit durch das Absetzen einer wirksamen Therapie [44] und einem drohenden Verlust an Lebensqualität geschildert. Eine vorgegebene begrenzte Therapiedauer mit CGRP/(R)-AK wird von Patienten oft als ein Eingriff in die Therapiefreiheit ihres Arztes empfunden. Das Erleben eines raschen Wirkeintritts zu Therapiebeginn mag die Erwartung eines raschen Wirkverlustes nach Absetzen der Behandlung beeinflussen und somit möglicherweise Noceboeffekte hervorrufen.

## Praxisfragen zum Beenden einer medikamentösen Migräneprophylaxe

### Frage 1

#### Ist das Beenden/Absetzen einer medikamentösen Migräneprophylaxe sinnvoll?

Variable Spontanverläufe, die generell günstige Prognose der Migräne sowie Daten zur anhaltenden Reduktion der MMDs bei einem Teil der Patienten nach Beenden der Migräneprophylaxe rechtfertigen es, die Indikation für eine medikamentöse Prophylaxe durch einen Absetzversuch zu prüfen. Dies gilt aufgrund der vorliegenden wissenschaftlichen Evidenz insbesondere für Patienten mit eM, kurzer Erkrankungsdauer und keinen oder gering ausgeprägten Komorbiditäten. Die bislang praktizierten Therapieempfehlungen erscheinen daher angemessen.

Vor allem bei Patienten mit einer hochfrequenten eM (≥ 8 MMDs) oder cM zu Beginn der Therapie muss aufgrund der vorgelegten Datenlage bei einem Absetzversuch eher mit einer Zunahme der klinischen Symptomatik und entsprechenden psychosozialen Folgen gerechnet werden als bei Patienten mit einer eM. Bei diesen Patienten ist es gerechtfertigt, die Migräneprophylaxe über einen Zeitraum von mehr als 12 Monaten fortzuführen. Das gilt insbesondere dann, wenn individuelle Konstellationen (z. B. psychische Belastungssituationen, bevorstehende soziale oder berufliche Veränderungen, ausgeprägte Angststörungen) ein hohes Risiko für eine erneute Zunahme der Migränefrequenz erwarten lassen. In Tab. 3 wurde anhand der aktuellen Literatur sowie basierend auf einem Expertenkonsensus eine Übersicht der Faktoren zusammengestellt, die für oder gegen einen Absetzversuch einer medikamentösen Migräneprophylaxe sprechen können.

**Table 3:** 

Argumente für Absetzversuch	Argumente gegen Absetzversuch
– Rasches und anhaltend gutes Ansprechen auf die Prophylaxe – < 8 MMDs und niedrige migränebedingte Beeinträchtigung vor Beginn der Therapie – Schlechte Verträglichkeit – Alter > 60 Jahre (prognostisch zu erwartende spontane Besserung der Migräne) – Wegfall von ungünstigen Begleitfaktoren (berufliche oder private Belastungsfaktoren) – Familienplanung	– Verzögertes Ansprechen auf die Prophylaxe – Gesicherter Wirknachweis, aber weiterhin hohe Krankheitslast (z. B. hohe Anzahl von MMDs, hohe Beeinträchtigung der Lebensqualität) – Hohe Anzahl von MMDs und hohe migränebedingte Beeinträchtigung vor Beginn der Therapie – Multiple vorangegangene unzureichend wirksame Therapieversuche der Migräneprophylaxe – Anhaltende und bevorstehende psychosoziale Belastungssituationen – Komplexe somatische und psychische Komorbiditäten – Kopfschmerz durch Medikamentenübergebrauch aktuell oder in der Vergangenheit – Bei Patienten unter Therapie mit OnabotulinumtoxinA- oder CGRP/(R)-AK-Therapie: wiederholt beobachtete nachlassende Wirkung zum Ende des Dosierungsintervalls

Unter Berücksichtigung häufiger Noceboeffekte kann anstelle des Absetzens die Verlängerung des Dosierintervalls als Dosisreduktion der CGRP/(R)-AK angewendet werden [45, 46]. Über das Vorgehen der Verlängerung des Dosierintervalls als Dosisreduktion, das bislang nicht in klinischen Studien untersucht wurde, sollten die Patienten aufgeklärt werden.

### Frage 2

#### Nach welcher Therapiedauer kann eine erfolgreiche Prophylaxe abgesetzt werden?

Die aktuelle Therapieleitlinie für Migräne empfiehlt, eine erfolgreiche Migräneprophylaxe nach 6–12 Monaten auf ihre Notwendigkeit zu überprüfen [6]. Wahrscheinlich ist aber eine Therapiedauer von 12 Monaten sinnvoller. Für klassische Migräneprophylaxen (Metoprolol, Propranolol, Amitriptylin, Flunarizin, Topiramat) wurden in Therapiestudien placebokontrolliert Effekte ab einem Therapiezeitraum von 4 Wochen nachgewiesen [12]. Die meisten Studien dieser Medikamente untersuchten Therapiezeiträume von 12 Wochen [12]. Das erlaubt jedoch keine Aussage, wie lange eine wirksame Migräneprophylaxe mindestens eingesetzt werden soll.

##### Besonderheiten bei der Behandlung mit OnabotulinumtoxinA.

OnabotulinumtoxinA ist in Deutschland und Österreich zur Behandlung der cM zugelassen. Da bisherige Daten zur Therapiedauer von OnabotulinumtoxinA-Injektionen bei cM unzureichend sind, stützt sich die Therapiedauer in Deutschland auf einen Expertenkonsens der DMKG für das strukturierte Vorgehen bei der Behandlung der cM [46].

In diesem werden während des ersten Therapiejahres Injektionsintervalle von 3 Monaten empfohlen. Ziel der Behandlung ist eine Verbesserung der Migräne um ≥ 30 %. Wird nach dem dritten Injektionszyklus keine Verbesserung um ≥ 30 % erreicht, gilt die Behandlung in der Regel als nicht ausreichend wirksam und es wird empfohlen, sie zu beenden. Wird im ersten Jahr der Behandlung ein stabiler Erfolg erreicht, kann eine Verlängerung der Injektionsintervalle auf 4 Monate in Betracht gezogen werden. Bleibt der Erfolg für mindestens zwei 4‑Monats-Intervalle stabil, kann versucht werden, die OnabotulinumtoxinA-Behandlung zu beenden, oder in Einzelfällen das Injektionsintervall auf 6 Monate verlängert werden.

##### Besonderheiten bei der Behandlung mit CGRP/(R)-AK.

Die Vorgaben zur Überprüfung einer Therapie bzw. eines Absetzens gemäß der Fachinformation fallen für die einzelnen Präparate und Zulassungsgebiete (EU und Schweiz) unterschiedlich aus (siehe Tab. 4).

**Table 4:** 

Substanz	EMA (D, A)	Swissmedic (CH)
Erenumab	Es wird empfohlen, nach den ersten drei Monaten der Behandlung in regelmäßigen Abständen zu evaluieren, ob die Behandlung fortzusetzen ist.	Bei mangelndem Therapieansprechen beziehungsweise nach spätestens 12 Monaten sollte eine Reevaluation zur Fortführung der Therapie vorgenommen werden.
Fremanezumab	Der Nutzen der Behandlung ist innerhalb von 3 Monaten nach Behandlungsbeginn zu bewerten. Jede weitere Entscheidung bezüglich einer Fortführung der Behandlung ist für jeden Patienten individuell abzuwägen. Es wird empfohlen, die Notwendigkeit zur Fortsetzung der Behandlung danach regelmäßig zu beurteilen.	Bei mangelndem Therapieansprechen beziehungsweise nach spätestens 12 Monaten, sollte eine Reevaluation zur Fortführung der Therapie vorgenommen werden.
Galcanezumab	Der Behandlungserfolg sollte 3 Monate nach Behandlungsbeginn beurteilt werden. Jede weitere Entscheidung, die Behandlung fortzusetzen, sollte für jeden Patienten individuell erfolgen. Es wird empfohlen, anschließend regelmäßig zu überprüfen, ob eine Weiterbehandlung notwendig ist.	Bei fehlendem Ansprechen auf die Therapie oder mindestens einmal pro Jahr sollte eine Reevaluation hinsichtlich Notwendigkeit der Fortführung der Therapie vorgenommen werden.
Eptinezumab	Der Nutzen und die Fortführung der Behandlung sollten 6 Monate nach Behandlungsbeginn beurteilt werden. Jede weitere Entscheidung über die Fortführung der Behandlung sollte für jeden Patienten individuell getroffen werden.	Bei mangelndem Therapieansprechen beziehungsweise nach spätestens 12 Monaten sollte eine Reevaluation zur Fortführung der Therapie vorgenommen werden.

Nach Empfehlung der Ergänzung der Therapieleitlinie für Migräne von 2019 [7] sollte die Therapie mit einem der CGRP/(R)-AKs, nachdem die Indikation gestellt wurde, zunächst für 3 Monate erfolgen. Wenn kein positiver Therapieeffekt (d. h. keine 50 % Reduktion der Migränetage, 30 % Reduktion MIDAS, 5 Punkte Reduktion HIT-6) besteht, sollte die Therapie beendet werden [7]. Bei Wirksamkeit der Therapie wird in der aktualisierten Leitlinie empfohlen, nach weiteren 9–12 Monaten zu überprüfen, ob die Therapie noch indiziert ist.

In Deutschland wurden bislang überwiegend Patienten mit einem CGRP/(R)-AK behandelt, die nicht auf empfohlene Erstlinienprophylaxen angesprochen haben oder diese aufgrund einer Kontraindikation bzw. nicht tolerierbarer Nebenwirkungen nicht einnehmen konnten [7]. Demzufolge handelte es sich hier häufig um Patienten mit einem prolongierten Krankheitsverlauf. Damit wird bei Patienten mit cM vom Gemeinsamen Bundesausschuss (GBA) eine Therapie mit Betablockern, Flunarizin oder Amitriptylin gefordert, deren Wirksamkeit bei der cM nie belegt wurde. Hier gibt es lediglich für Propranolol (160 mg/Tag) erste Daten, dass die Therapieeffekte auf MMD und Alltagsfähigkeit bei Patienten mit chronischer Migräne vergleichbar mit Topiramat (100 mg/Tag) sind [47].

Wenn für diese Patienten aufgrund der Therapie mit einem CGRP/(R)-AK eine deutliche Reduktion der MMDs, der Analgetikatage und/oder der migränebedingten Alltagseinschränkung erreicht wird, dann hat das neben der biologischen Wirkung des Medikaments eine enorme psychische und soziale Implikation für den Alltag der Patienten. So konnte für Patienten mit begleitender Depression unter der Therapie mit CGRP/(R)-AK eine Verbesserung der migränebedingten Einschränkung und eine globale Verbesserung der Gesundheitssituation gezeigt werden [48]. Auch wurde unter einer wirkungsvollen Therapie mit CGRP/(R)-AK eine Verbesserung der Arbeitsfähigkeit und Teilnahme am sozialen Leben nachgewiesen [49]. Nicht zuletzt wurde die Inanspruchnahme des Gesundheitssystems mit Notfallvorstellungen, Krankenhausaufnahmen, Diagnostik und Arztkonsultationen verringert [50].

Die Wahl des Zeitpunkts für einen Absetzversuch einer Migräneprophylaxe bleibt daher immer eine Einzelfallentscheidung des behandelnden Arztes gemeinsam mit dem Patienten unter Berücksichtigung der medizinischen, psychischen und der aktuellen sozialen Situation des Patienten, d. h. der individuellen Krankheitslast bestehend aus Alter, vorhandenen migränebedingten Einschränkungen, Bedarf an Akutmedikation und Komorbiditäten sowie bisherigem Ansprechen auf die aktuelle Therapie (siehe auch Tab. 3). Dementsprechend kann bei Patienten mit niedrigfrequenter (< 8 MMDs) eM zu Beginn der Prophylaxe und kürzerer Erkrankungsdauer sowie fehlenden Komorbiditäten die Einnahme einer effektiven Prophylaxe für 6 bis 12 Monate ausreichend sein. Dagegen benötigen Patienten mit längerer Migräneanamnese, hochfrequenter eM (≥ 8 MMDs) oder cM zu Behandlungsbeginn und vorliegenden relevanten Komorbiditäten mindestens 12 Monate, in Einzelfällen auch längere Therapiedauer, bevor ein Absetzen medizinisch sinnvoll ist. Hintergrund ist, dass diese vulnerable Patientengruppe aufgrund umfangreicher Chronifizierungsprozesse mit Remodelling des zentralnervösen Schmerznetzwerks nachhaltig angelegte, also längerfristige Therapiekonzepte braucht [51]. Bei Patienten mit einem Medikamentenübergebrauch in der Anamnese wird wegen des erheblichen Risikos eines erneuten MOH auch bisher bereits empfohlen, prophylaktische Maßnahmen für mindestens 2 Jahre fortzuführen. Dies sollte auch bei einer Therapie mit einem CGRP/(R)-AK in gleicher Weise berücksichtigt werden [6].

Die aktuelle Leitlinie der European Headache Foundation zum Einsatz von CGRP/(R)-AKs bei eM und cM sieht ein Absetzen der CGRP/(R)-AKs nach 12–18 Monaten vor. Allerdings wird darauf verwiesen, dass bei Bedarf die Behandlung so lange wie nötig fortgesetzt werden kann [52].

### Frage 3

#### Welche Kriterien gelten für die Wiederaufnahme einer medikamentösen Prophylaxe?

Die Indikationen zum Beginn einer medikamentösen Migräneprophylaxe sind in Tab. 1 zusammengefasst. Die Kriterien gelten auch für die Wiederaufnahme einer medikamentösen Prophylaxe nach dem Absetzen. In der Regel wird man die Behandlung wieder mit der Substanz durchführen, die in der Vergangenheit einen guten Effekt gezeigt hat und vertragen wurde. Grund dafür ist, dass bei den unspezifischen Prophylaxen eine Umstellung der Prophylaxe mit einer niedrigeren Therapietreue einhergeht [53]. Die wenigen bisher publizierten Daten zu CGRP/(R)-AK deuten darauf hin, dass die Anwendung auch nach einem Wechsel zu einem anderen CGRP/(R)-AK („Switch“ des Antikörpers) mit hoher Therapietreue fortgesetzt wird [54]. Die aktuelle Richtlinie der European Headache Foundation empfiehlt, die Behandlung mit CGRP/(R)-AK wieder aufzunehmen, wenn die Zahl der MMDs nach Absetzen der CGRP/(R)-AK-Behandlung erneut zunimmt [52].

In jedem Fall ist eine verlässliche Dokumentation der Migränefrequenz, der Kopfschmerztage und des Bedarfs an Akutmedikation erforderlich. Dazu sollten Daten aus einem geeigneten Kopfschmerzkalender (Papier- oder App-basiert, z. B. über die DMKG-App) und/oder Fragebögen wie HIT‑6 oder MIDAS genutzt werden.

### Frage 4

#### Was muss bei einer langfristig eingesetzten medikamentösen Migräneprophylaxe beachtet werden?

Wie bei jeder medikamentösen Therapie muss während der Behandlung auf die Entwicklung von Komorbiditäten, die eine Kontraindikation der Medikation bedingen können, sowie Wechselwirkungen mit anderen Medikamenten geachtet werden. Kontrolluntersuchungen von Laborparametern und EKG sind wie in den Fachinformationen empfohlen durchzuführen. Klinische Verlaufskontrollen sind auch bei guter Verträglichkeit einer Substanz zu empfehlen. Dies gilt insbesondere bei neuen Substanzklassen wie den CGRP/(R)-AK, da hier noch keine umfangreichen Langzeiterfahrungen vorliegen. In die bisherigen Studien mit CGRP/(R)-AKs wurden nur wenige ältere Patienten (≥ 65 Jahre) eingeschlossen, somit ist hier bei einer Langzeitbehandlung besondere Sorgfalt geboten. Die CGRP/(R)-AK sind derzeit bei Kindern nicht zugelassen.

Aufgrund des pathophysiologischen Wirkmechanismus der CGRP/(R)-AK sollten Patienten mit erhöhtem zerebro- und kardiovaskulärem Risiko, entzündlichen Darmerkrankungen, COPD und Wundheilungsstörungen nur unter strenger Nutzen-Risiko-Bewertung auf diese Substanzklasse eingestellt werden. Regelmäßige Verlaufskontrollen der Patienten unter besonderer Beachtung der Begleiterkrankung sind erforderlich. Eine Übersicht der im klinischen Alltag häufig auftretenden Kontraindikationen medikamentöser Migräneprophylaxen zeigt Tab. 5.

**Table 5:** 

Migräneprophylaxe	Kontraindikation^a^	Bemerkung
Amitriptylin	Herzinfarkt Koronare Herzkrankheit Herzrhythmusstörungen Schwere Lebererkrankung	Kontrollen von Labor (Blutbild, Leberfunktionstest), EKG
Metoprolol/Propranolol	Herzinsuffizienz, AV-Block 2. oder 3. Grades, Bradykardie, Hypotonie	Kontrollen von Blutzucker bei Diabetikern, EKG
Topiramat	Keine hochwirksame Verhütungsmethode, Schwangerschaft Nephrolithiasis	Cave Einfluss auf orale Kontrazeption bei > 200 mg/Tag
Flunarizin	Extrapyramidalen Erkrankungen, depressives Syndrom Schwangerschaft	Sedierende Wirkung kann durch Alkohol, Schlafmittel, Tranquilizer verstärkt werden
OnabotulinumtoxinA	Neuromuskuläre Erkrankungen, Schwangerschaft und Stillzeit	In Deutschland: Zulassung für chronische Migräne ab 18. Lebensjahr
Erenumab Galcanezumab Fremanezumab Eptinezumab	Schwangerschaft, Stillzeit Einsatz bei erhöhtem zerebro-/kardiovaskulärem Risiko, Raynaud-Syndrom, entzündlichen Darmerkrankungen, COPD, Wundheilungsstörungen unter strenger Nutzen-Risiko-Bewertung	Zur Sicherheit bei Patienten > 65 Jahre liegen nur einzelne kleine Fallserien vor

*BZ* Blutzucker

^a^Auswahl häufiger Kontraindikationen

## Supplementary Information




