# Erratum zu: Konsensusstatement der Migräne- und Kopfschmerzgesellschaften (DMKG, ÖKSG & SKG) zur Therapiedauer der medikamentösen Migräneprophylaxe

**DOI:** 10.1007/s00482-022-00691-5

**Published:** 2023-01-20

**Authors:** Gudrun Goßrau, Stefanie Förderreuther, Ruth Ruscheweyh, Victoria Ruschil, Till Sprenger, David Lewis, Katharina Kamm, Tobias Freilinger, Lars Neeb, Volker Malzacher, Uwe Meier, Klaus Gehring, Torsten Kraya, Thomas Dresler, Christoph J. Schankin, Andreas R. Gantenbein, Gregor Brössner, Karin Zebenholzer, Hans-Christoph Diener, Charly Gaul, Tim P. Jürgens

**Affiliations:** 1 Kopfschmerzambulanz, Universitätsschmerzcentrum, Medizinische Fakultät der TU Dresden, Universitätsklinikum Dresden, Fetscherstr. 74, 01307 Dresden, Deutschland; 2 Neurologische Klinik und Poliklinik, Ludwig-Maximilians-Universität München, München, Deutschland; 3 Deutsche Migräne- und Kopfschmerzgesellschaft, Frankfurt, Deutschland; 4 Klinik für Psychosomatik und Psychotherapie, Technische Universität München, München, Deutschland; 5 Abteilung Neurologie mit Schwerpunkt Epileptologie, Universitätsklinikum Tübingen, Tübingen, Deutschland; 6 Deutsche Klinik für Diagnostik, DKD Helios Klinik Wiesbaden, Wiesbaden, Deutschland; 7 LEWIS Neurologie, Stuttgart, Deutschland; 8 Klinik für Neurologie, Klinikum Passau, Passau, Deutschland; 9 Helios Global Health, Berlin, Deutschland; 10 Charité – Universitätsmedizin Berlin, corporate member of Freie Universität Berlin and Humboldt Universität zu Berlin, Klinik und Hochschulambulanz für Neurologie, Berlin, Deutschland; 11 Neurozentrum Reutlingen, Reutlingen, Deutschland; 12 Berufsverband Deutscher Neurologen, Wulffstr. 8, 12165 Berlin, Deutschland; 13 Berufsverband Deutscher Nervenärzte, Wulffstr. 8, 12165 Berlin, Deutschland; 14 Neurologische Klinik, Krankenhaus Sankt Georg Leipzig, Leipzig, Deutschland; 15 Neurologische Klinik, Universitätsklinikum Halle-Saale, Halle-Saale, Deutschland; 16 Klinik für Psychiatrie und Psychotherapie, Tübingen Zentrum für seelische Gesundheit, Universitätsklinikum Tübingen, Tübingen, Deutschland; 17 LEAD Graduiertenschule & Forschungsnetzwerk, Universität Tübingen, Tübingen, Deutschland; 18 Neurologische Klinik, Inselspital, Universitätsspital Bern, Universität Bern, Bern, Schweiz; 19 Universitätsspital Bern, Universität Bern, Bern, Schweiz; 20 Neurologie & Schmerz, ZURZACH Care, Bad Zurzach, Schweiz; 21 Praxis Neurologie am Untertor, Bülach, Schweiz; 22 Universitätsklinik für Neurologie, Medizinische Universität Innsbruck, Innsbruck, Österreich; 23 Universitätsklinik für Neurologie, Medizinische Universität Wien, Wien, Österreich; 24 Institut für Medizinische Informatik, Biometrie und Epidemiologie (IMIBE), Medizinische Fakultät, Universität Duisburg-Essen, Essen, Deutschland; 25 Kopfschmerzzentrum Frankfurt, Frankfurt, Deutschland; 26 Kopfschmerzzentrum Nordost, Neurologische Klinik und Poliklinik, Universitätsklinik Rostock, Rostock, Deutschland; 27 Neurologische Klinik, KMG Krankenhaus Güstrow, Güstrow, Deutschland

**Erratum zu:**

**Schmerz 2022**

10.1007/s00482-022-00671-9

In diesem Artikel waren die Adressdaten für den Autor Lars Neeb falsch angegeben. Statt „Neurologische Klinik und Poliklinik, Institut für Public Health, Charité-Universitätsmedizin Berlin, Freie Universität Berlin und Humboldt Universität zu Berlin, Berlin, Deutschland“ hätten sie „Charité – Universitätsmedizin Berlin, corporate member of Freie Universität Berlin and Humboldt Universität zu Berlin, Klinik und Hochschulambulanz für Neurologie, Berlin, Deutschland“ lauten sollen.

Der Originalbeitrag wurde korrigiert.

